# (2*E*)-1-(2,6-Dichloro-3-fluoro­phen­yl)-3-phenyl­prop-2-en-1-one

**DOI:** 10.1107/S1600536812010574

**Published:** 2012-03-17

**Authors:** Aletti S. Praveen, Hemmige S. Yathirajan, Badiadka Narayana, Thomas Gerber, Eric Hosten, Richard Betz

**Affiliations:** a University of Mysore, Department of Studies in Chemistry, Manasagangotri, Mysore 570 006, India; b Mangalore University, Department of Studies in Chemistry, Mangalagangotri 574 199, India; c Nelson Mandela Metropolitan University, Summerstrand Campus, Department of Chemistry, University Way, Summerstrand, PO Box 77000, Port Elizabeth 6031, South Africa

## Abstract

In the title compound, C_15_H_9_Cl_2_FO, the F atom shows positional disorder over two positions, with site-occupancy factors of 0.747 (4) and 0.253 (4). The dihedral angle between the rings is 86.37 (10)°. In the crystal, C—H⋯O contacts connect the mol­ecules into chains along the *c* axis. The shortest inter-centroid distance between two aromatic systems is 3.6686 (12) Å and is apparent between the halogenated rings.

## Related literature

For pharmaceutical background to chalcones, see: Lin *et al.* (2002 ▶); Modzelewska *et al.* (2006 ▶); Svetaz *et al.* (2004 ▶). For related structures, see: Betz *et al.* (2012 ▶). For graph-set analysis of hydrogen bonds, see: Etter *et al.* (1990 ▶); Bernstein *et al.* (1995 ▶).
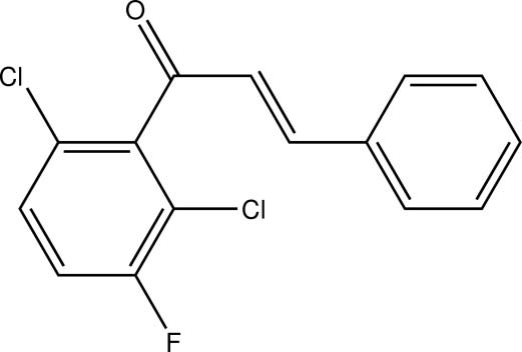


## Experimental

### 

#### Crystal data

C_15_H_9_Cl_2_FO
*M* *_r_* = 295.12
Monoclinic, 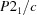

*a* = 11.3390 (3) Å
*b* = 10.3896 (3) Å
*c* = 11.3930 (3) Å
β = 97.078 (1)°
*V* = 1331.95 (6) Å^3^
*Z* = 4
Mo *K*α radiation
μ = 0.49 mm^−1^
*T* = 200 K
0.49 × 0.34 × 0.17 mm

#### Data collection

Bruker APEXII CCD diffractometer
Absorption correction: multi-scan (*SADABS*; Bruker, 2008 ▶) *T*_min_ = 0.798, *T*_max_ = 0.920
23002 measured reflections
3329 independent reflections
2742 reflections with *I* > 2σ(*I*)
*R*_int_ = 0.025

#### Refinement

*R*[*F* ^2^ > 2σ(*F* ^2^)] = 0.043
*wR*(*F* ^2^) = 0.111
*S* = 1.04
3329 reflections
182 parameters
H-atom parameters constrained
Δρ_max_ = 0.58 e Å^−3^
Δρ_min_ = −0.60 e Å^−3^

### 

Data collection: *APEX2* (Bruker, 2010 ▶); cell refinement: *SAINT* (Bruker, 2010 ▶); data reduction: *SAINT*; program(s) used to solve structure: *SHELXS97* (Sheldrick, 2008 ▶); program(s) used to refine structure: *SHELXL97* (Sheldrick, 2008 ▶); molecular graphics: *ORTEP-3* (Farrugia, 1997 ▶) and *Mercury* (Macrae *et al.*, 2008 ▶); software used to prepare material for publication: *SHELXL97* and *PLATON* (Spek, 2009 ▶).

## Supplementary Material

Crystal structure: contains datablock(s) I, global. DOI: 10.1107/S1600536812010574/hg5184sup1.cif

Supplementary material file. DOI: 10.1107/S1600536812010574/hg5184Isup2.cdx

Structure factors: contains datablock(s) I. DOI: 10.1107/S1600536812010574/hg5184Isup3.hkl

Supplementary material file. DOI: 10.1107/S1600536812010574/hg5184Isup4.cml

Additional supplementary materials:  crystallographic information; 3D view; checkCIF report

## Figures and Tables

**Table 1:** Hydrogen-bond geometry (Å, °)

*D*—H⋯*A*	*D*—H	H⋯*A*	*D*⋯*A*	*D*—H⋯*A*
C1—H1⋯O1^i^	0.95	2.50	3.399 (2)	158
C16—H16⋯O1^i^	0.95	2.57	3.440 (2)	153

Symmetry code: (i) 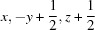.

**Fig. 1.**
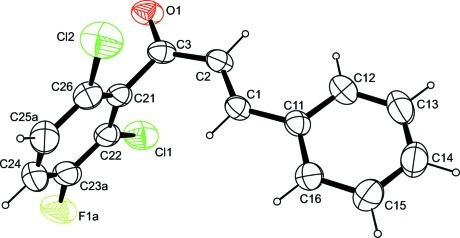
The molecular structure of the title compound, with atom labels and anisotropic displacement ellipsoids (drawn at 50% probability level).

**Fig. 2.**
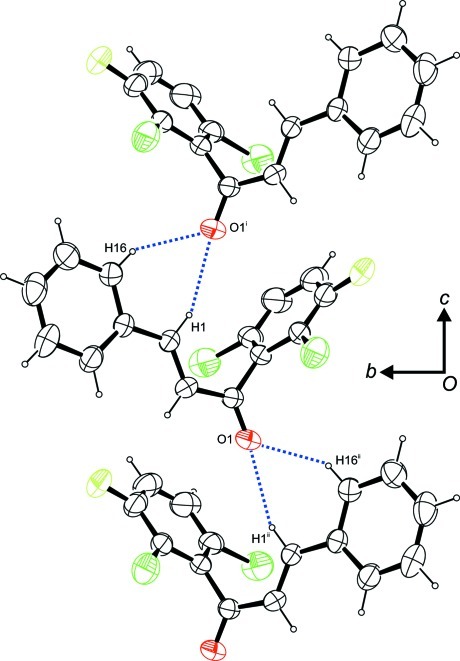
Intermolecular contacts, viewed along [1 0 0]. Symmetry operators: ix, -y + 1/2, z + 1/2; iix, -y + 1/2, z - 1/2.

**Fig. 3.**
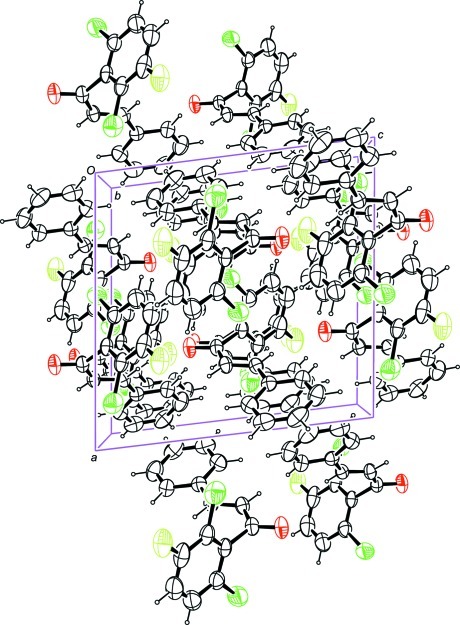
Molecular packing of the title compound, viewed along [0 1 0] (anisotropic displacement ellipsoids drawn at 50% probability level).

## supplementary crystallographic information

### Comment

Substituted chalcones and their derivatives have been reported to possess interesting biological properties such as being antitubercular (Lin *et al.*, 2002), anticancer (Modzelewska *et al.*, 2006) and antifungal agents (Svetaz *et al.*, 2004). The crystal structures of some chalcones such as (2*E*)-1-(2,6-Dichloro-3-fluorophenyl)-3-(4-fluorophenyl)prop-2-en-1-one (Betz *et al.*, 2012) have been reported in the literature. As part of our ongoing studies on chalcones, the title compound was synthesized and characterized by X-ray diffraction.

The fluorine atom on the halogenated phenyl ring shows rotational disorder over two positions with site occupancy factors of 0.75 and 0.25. The least-squares planes defined by the carbon atoms of the two aromatic moieties intersect at an angle of 86.37 (10)° (Fig. 1).

In the crystal, C–H···O contacts whose range falls by more than 0.1 Å below the sum of van-der-Waals radii of the respective atoms are present. These are supported by one of the vinylic hydrogen atoms and one of the hydrogen atoms of the unsubstituted phenyl ring. In terms of graph-set analysis (Etter *et al.*, 1990; Bernstein *et al.*, 1995), the descriptor for the hydrogen bonds is *C*^1^_1_(5)*C*^1^_1_(7) on the unary level. Metrical information about these contacts as well as their symmetry is summarized in Table 1. In total, the molecules are connected to chains along the crystallographic *c* axis. The shortest intercentroid distance between two aromtic systems was measured at 3.6686 (12) Å and is apparent between the halogenated phenyl rings (Fig. 2).

The packing of the title compound in the crystal structure is shown in Figure 3.

### Experimental

To a stirred solution of 1-(2,6-dichloro-3-fluorophenyl)ethanone (1 g, 4.8 mmol) and benzaldehyde (0.51 g, 4.8 mmol) in ethanol (10 ml), powdered KOH (0.40 g 7.2 mmol) was added at 0 °C. The reaction mixture was stirred at room temperature for 2 h. After completion of the reaction, the mixture was pourred into ice cold water and subsequently acidified with 1.5 N HCl (pH ~3). The precipitated solid was filtered and dried to afford 1.2 g of the title compound as off-white solid in 86% yield. The single-crystal was grown from a mixture of toluene:acetone (*v*:*v* = 1:1) by slow evaporation at room temperature (m.p.: 385–388 K).

### Refinement

Carbon-bound H atoms were placed in calculated positions (C—H 0.95 Å) and were included in the refinement in the riding model approximation, with *U*(H) set to 1.2*U*_eq_(C).

### Crystal data

**Table:**

C_15_H_9_Cl_2_FO	*F*(000) = 600
*M**_r_* = 295.12	*D*_x_ = 1.472 Mg m^−^^3^
Monoclinic, *P*2_1_/*c*	Melting point = 385–388 K
Hall symbol: -P 2ybc	Mo *K*α radiation, λ = 0.71073 Å
*a* = 11.3390 (3) Å	Cell parameters from 9971 reflections
*b* = 10.3896 (3) Å	θ = 2.7–28.3°
*c* = 11.3930 (3) Å	µ = 0.49 mm^−^^1^
β = 97.078 (1)°	*T* = 200 K
*V* = 1331.95 (6) Å^3^	Block, colourless
*Z* = 4	0.49 × 0.34 × 0.17 mm

### Data collection

**Table:**

Bruker APEXII CCD diffractometer	3329 independent reflections
Radiation source: fine-focus sealed tube	2742 reflections with *I* > 2σ(*I*)
Graphite monochromator	*R*_int_ = 0.025
φ and ω scans	θ_max_ = 28.4°, θ_min_ = 2.7°
Absorption correction: multi-scan (*SADABS*; Bruker, 2008)	*h* = −15→15
*T*_min_ = 0.798, *T*_max_ = 0.920	*k* = −13→13
23002 measured reflections	*l* = −15→15

### Refinement

**Table:**

Refinement on *F*^2^	Primary atom site location: structure-invariant direct methods
Least-squares matrix: full	Secondary atom site location: difference Fourier map
*R*[*F*^2^ > 2σ(*F*^2^)] = 0.043	Hydrogen site location: inferred from neighbouring sites
*wR*(*F*^2^) = 0.111	H-atom parameters constrained
*S* = 1.04	*w* = 1/[σ^2^(*F*_o_^2^) + (0.040*P*)^2^ + 0.7737*P*] where *P* = (*F*_o_^2^ + 2*F*_c_^2^)/3
3329 reflections	(Δ/σ)_max_ = 0.001
182 parameters	Δρ_max_ = 0.58 e Å^−^^3^
0 restraints	Δρ_min_ = −0.60 e Å^−^^3^

### Fractional atomic coordinates and isotropic or equivalent isotropic displacement parameters (Å^2^)

**Table:**

	*x*	*y*	*z*	*U*_iso_*/*U*_eq_	Occ. (<1)
Cl1	0.83699 (6)	0.03537 (6)	0.06241 (5)	0.06914 (19)	
Cl2	0.43775 (5)	0.32132 (6)	−0.01452 (5)	0.07146 (19)	
O1	0.67486 (13)	0.20446 (13)	−0.16536 (10)	0.0526 (3)	
C1	0.76748 (14)	0.40662 (15)	0.08071 (14)	0.0373 (3)	
H1	0.7325	0.3570	0.1374	0.045*	
C2	0.75116 (16)	0.36328 (16)	−0.03056 (14)	0.0410 (4)	
H2	0.7832	0.4114	−0.0901	0.049*	
C3	0.68640 (16)	0.24564 (16)	−0.06433 (13)	0.0399 (3)	
C11	0.83272 (14)	0.52106 (15)	0.12542 (14)	0.0373 (3)	
C12	0.89663 (17)	0.59959 (17)	0.05617 (17)	0.0486 (4)	
H12	0.8986	0.5798	−0.0250	0.058*	
C13	0.95691 (18)	0.70569 (19)	0.1055 (2)	0.0569 (5)	
H13	1.0018	0.7574	0.0584	0.068*	
C14	0.9526 (2)	0.7373 (2)	0.2222 (2)	0.0622 (6)	
H14	0.9937	0.8112	0.2552	0.075*	
C15	0.8889 (2)	0.6619 (2)	0.29125 (19)	0.0602 (5)	
H15	0.8850	0.6843	0.3715	0.072*	
C16	0.83055 (16)	0.55359 (17)	0.24359 (15)	0.0448 (4)	
H16	0.7884	0.5007	0.2922	0.054*	
C21	0.63217 (16)	0.17125 (15)	0.03024 (13)	0.0407 (4)	
C22	0.69437 (18)	0.07128 (16)	0.09046 (15)	0.0467 (4)	
C24	0.5310 (2)	0.0283 (2)	0.20019 (19)	0.0693 (7)	
H24	0.4966	−0.0210	0.2574	0.083*	
C26	0.51877 (18)	0.19772 (19)	0.05775 (16)	0.0492 (4)	
C23A	0.6426 (2)	0.00145 (19)	0.17485 (17)	0.0618 (6)	0.747 (4)
C25A	0.4689 (2)	0.1274 (3)	0.14214 (19)	0.0665 (7)	0.747 (4)
H25A	0.3913	0.1478	0.1599	0.080*	0.747 (4)
F1A	0.7017 (2)	−0.09064 (16)	0.23597 (15)	0.0758 (7)	0.747 (4)
C23B	0.6426 (2)	0.00145 (19)	0.17485 (17)	0.0618 (6)	0.25
H23B	0.6862	−0.0665	0.2157	0.074*	0.253 (4)
C25B	0.4689 (2)	0.1274 (3)	0.14214 (19)	0.0665 (7)	0.25
F2B	0.3774 (5)	0.1417 (8)	0.1629 (6)	0.099 (3)	0.253 (4)

### Atomic displacement parameters (Å^2^)

**Table:**

	*U*^11^	*U*^22^	*U*^33^	*U*^12^	*U*^13^	*U*^23^
Cl1	0.0789 (4)	0.0587 (3)	0.0676 (3)	0.0216 (3)	−0.0001 (3)	0.0108 (2)
Cl2	0.0627 (3)	0.0829 (4)	0.0700 (4)	0.0201 (3)	0.0129 (3)	0.0075 (3)
O1	0.0774 (9)	0.0498 (7)	0.0311 (6)	0.0050 (6)	0.0081 (6)	−0.0060 (5)
C1	0.0450 (8)	0.0320 (7)	0.0358 (7)	0.0024 (6)	0.0077 (6)	0.0041 (6)
C2	0.0523 (9)	0.0386 (8)	0.0327 (7)	0.0025 (7)	0.0077 (6)	0.0057 (6)
C3	0.0512 (9)	0.0373 (8)	0.0311 (7)	0.0084 (7)	0.0051 (6)	0.0000 (6)
C11	0.0393 (8)	0.0319 (7)	0.0403 (8)	0.0043 (6)	0.0031 (6)	0.0045 (6)
C12	0.0544 (10)	0.0406 (9)	0.0516 (10)	0.0008 (8)	0.0098 (8)	0.0096 (7)
C13	0.0501 (10)	0.0430 (9)	0.0763 (14)	−0.0047 (8)	0.0025 (9)	0.0187 (9)
C14	0.0637 (12)	0.0423 (10)	0.0743 (14)	−0.0108 (9)	−0.0161 (10)	0.0041 (9)
C15	0.0760 (14)	0.0490 (11)	0.0522 (11)	−0.0091 (10)	−0.0054 (10)	−0.0057 (9)
C16	0.0537 (10)	0.0392 (8)	0.0409 (8)	−0.0021 (7)	0.0027 (7)	0.0004 (7)
C21	0.0568 (10)	0.0352 (8)	0.0291 (7)	−0.0053 (7)	0.0009 (6)	−0.0041 (6)
C22	0.0665 (11)	0.0360 (8)	0.0354 (8)	−0.0068 (8)	−0.0030 (7)	−0.0018 (6)
C24	0.0934 (17)	0.0662 (14)	0.0480 (11)	−0.0410 (13)	0.0068 (11)	0.0041 (10)
C26	0.0567 (10)	0.0520 (10)	0.0385 (8)	−0.0074 (8)	0.0036 (7)	−0.0054 (7)
C23A	0.0988 (18)	0.0423 (10)	0.0404 (10)	−0.0211 (10)	−0.0078 (10)	0.0058 (8)
C25A	0.0706 (15)	0.0780 (15)	0.0518 (11)	−0.0294 (12)	0.0114 (10)	−0.0063 (11)
F1A	0.1208 (17)	0.0477 (9)	0.0571 (10)	0.0024 (9)	0.0037 (10)	0.0182 (7)
C23B	0.0988 (18)	0.0423 (10)	0.0404 (10)	−0.0211 (10)	−0.0078 (10)	0.0058 (8)
C25B	0.0706 (15)	0.0780 (15)	0.0518 (11)	−0.0294 (12)	0.0114 (10)	−0.0063 (11)
F2B	0.066 (4)	0.136 (6)	0.103 (5)	−0.033 (4)	0.045 (3)	−0.012 (4)

### Geometric parameters (Å, º)

**Table:**

Cl1—C22	1.727 (2)	C14—C15	1.376 (3)
Cl2—C26	1.728 (2)	C14—H14	0.9500
O1—C3	1.2198 (19)	C15—C16	1.382 (3)
C1—C2	1.337 (2)	C15—H15	0.9500
C1—C11	1.459 (2)	C16—H16	0.9500
C1—H1	0.9500	C21—C26	1.388 (3)
C2—C3	1.453 (2)	C21—C22	1.388 (2)
C2—H2	0.9500	C22—C23A	1.391 (3)
C3—C21	1.516 (2)	C24—C23A	1.362 (4)
C11—C16	1.391 (2)	C24—C25A	1.370 (4)
C11—C12	1.398 (2)	C24—H24	0.9500
C12—C13	1.380 (3)	C26—C25A	1.383 (3)
C12—H12	0.9500	C23A—F1A	1.317 (3)
C13—C14	1.377 (3)	C25A—H25A	0.9500
C13—H13	0.9500		
			
C2—C1—C11	127.65 (15)	C16—C15—H15	120.0
C2—C1—H1	116.2	C15—C16—C11	120.87 (18)
C11—C1—H1	116.2	C15—C16—H16	119.6
C1—C2—C3	122.74 (15)	C11—C16—H16	119.6
C1—C2—H2	118.6	C26—C21—C22	117.70 (16)
C3—C2—H2	118.6	C26—C21—C3	121.83 (15)
O1—C3—C2	122.47 (16)	C22—C21—C3	120.45 (16)
O1—C3—C21	119.07 (15)	C21—C22—C23A	120.0 (2)
C2—C3—C21	118.46 (13)	C21—C22—Cl1	120.01 (14)
C16—C11—C12	118.46 (16)	C23A—C22—Cl1	119.97 (16)
C16—C11—C1	117.86 (15)	C23A—C24—C25A	119.2 (2)
C12—C11—C1	123.68 (15)	C23A—C24—H24	120.4
C13—C12—C11	120.13 (18)	C25A—C24—H24	120.4
C13—C12—H12	119.9	C25A—C26—C21	121.5 (2)
C11—C12—H12	119.9	C25A—C26—Cl2	119.02 (18)
C14—C13—C12	120.62 (19)	C21—C26—Cl2	119.52 (14)
C14—C13—H13	119.7	F1A—C23A—C24	117.6 (2)
C12—C13—H13	119.7	F1A—C23A—C22	120.9 (2)
C15—C14—C13	119.97 (19)	C24—C23A—C22	121.5 (2)
C15—C14—H14	120.0	C24—C25A—C26	120.2 (2)
C13—C14—H14	120.0	C24—C25A—H25A	119.9
C14—C15—C16	119.9 (2)	C26—C25A—H25A	119.9
C14—C15—H15	120.0		
			
C11—C1—C2—C3	178.53 (15)	C26—C21—C22—C23A	0.1 (2)
C1—C2—C3—O1	−177.72 (17)	C3—C21—C22—C23A	−178.32 (15)
C1—C2—C3—C21	1.8 (2)	C26—C21—C22—Cl1	−178.77 (13)
C2—C1—C11—C16	175.07 (17)	C3—C21—C22—Cl1	2.8 (2)
C2—C1—C11—C12	−4.7 (3)	C22—C21—C26—C25A	0.0 (3)
C16—C11—C12—C13	0.8 (3)	C3—C21—C26—C25A	178.42 (17)
C1—C11—C12—C13	−179.44 (16)	C22—C21—C26—Cl2	179.90 (12)
C11—C12—C13—C14	−1.6 (3)	C3—C21—C26—Cl2	−1.7 (2)
C12—C13—C14—C15	0.8 (3)	C25A—C24—C23A—F1A	177.08 (19)
C13—C14—C15—C16	0.8 (3)	C25A—C24—C23A—C22	−0.8 (3)
C14—C15—C16—C11	−1.6 (3)	C21—C22—C23A—F1A	−177.52 (17)
C12—C11—C16—C15	0.8 (3)	Cl1—C22—C23A—F1A	1.4 (3)
C1—C11—C16—C15	−178.99 (17)	C21—C22—C23A—C24	0.3 (3)
O1—C3—C21—C26	−92.4 (2)	Cl1—C22—C23A—C24	179.16 (16)
C2—C3—C21—C26	88.0 (2)	C23A—C24—C25A—C26	0.9 (3)
O1—C3—C21—C22	85.9 (2)	C21—C26—C25A—C24	−0.5 (3)
C2—C3—C21—C22	−93.60 (19)	Cl2—C26—C25A—C24	179.59 (16)

### Hydrogen-bond geometry (Å, º)

**Table:**

*D*—H···*A*	*D*—H	H···*A*	*D*···*A*	*D*—H···*A*
C1—H1···O1^i^	0.95	2.50	3.399 (2)	158
C16—H16···O1^i^	0.95	2.57	3.440 (2)	153

Symmetry code: (i) *x*, −*y*+1/2, *z*+1/2.
